# Funnel chest (pectus excavatum): a rare clinical image

**DOI:** 10.11604/pamj.2023.45.194.40769

**Published:** 2023-08-31

**Authors:** Minal Dambhare, Bibin Kurian

**Affiliations:** 1 Department of Pediatric Nursing Smt. Radhikabai Meghe Memorial Memorial College of Nursing Datta Meghe Institute of Higher Education and Research (Deemed University) Sawangi Wardha, Maharashtra, India

**Keywords:** Pectus excavatum, funnel chest, Nuss procedure, surgical correction, clinical image

## Image in medicine

Congenital chest wall abnormality called “pectus excavatum,” or “funnel chest” is characterized by an inward dip of the sternum and surrounding ribs. In this condition, the breastbone is sunken inward, creating a concave or “funnel-like” appearance on the front of the chest. Pectus excavatum is the most congenital prevalent chest wall deformity, affecting one in 300-400 people. The condition is more prevalent in males and often becomes noticeable during early childhood or adolescence, although the precise cause of funnel chest is unknown, it is believed to involve a combination of genetic and environmental factors. The Nuss procedure is a widely accepted surgical technique for the correction of severe pectus excavatum. A 16-year-old male presented to our clinic with a noticeable depression in the center of his chest and complaints of exercise intolerance and shortness of breath. A physical examination is usually sufficient to diagnose funnel chest. The physical examination results showed a severe pectus excavatum with a Haller index of 4.5. Tests of pulmonary function revealed a pattern of restriction with decreased forced vital capacity (FVC) and forced expiratory volume in one second (FEV1). In severe cases, imaging procedures like X-rays, computed tomography scans, or magnetic resonance imaging are utilized to assess the severity of the situation and determine any associated consequences on the heart and lungs. Certain exercises and physical therapy techniques can help improve posture, strengthen the chest muscles, and potentially minimize the appearance of pectus.

**Figure 1:**
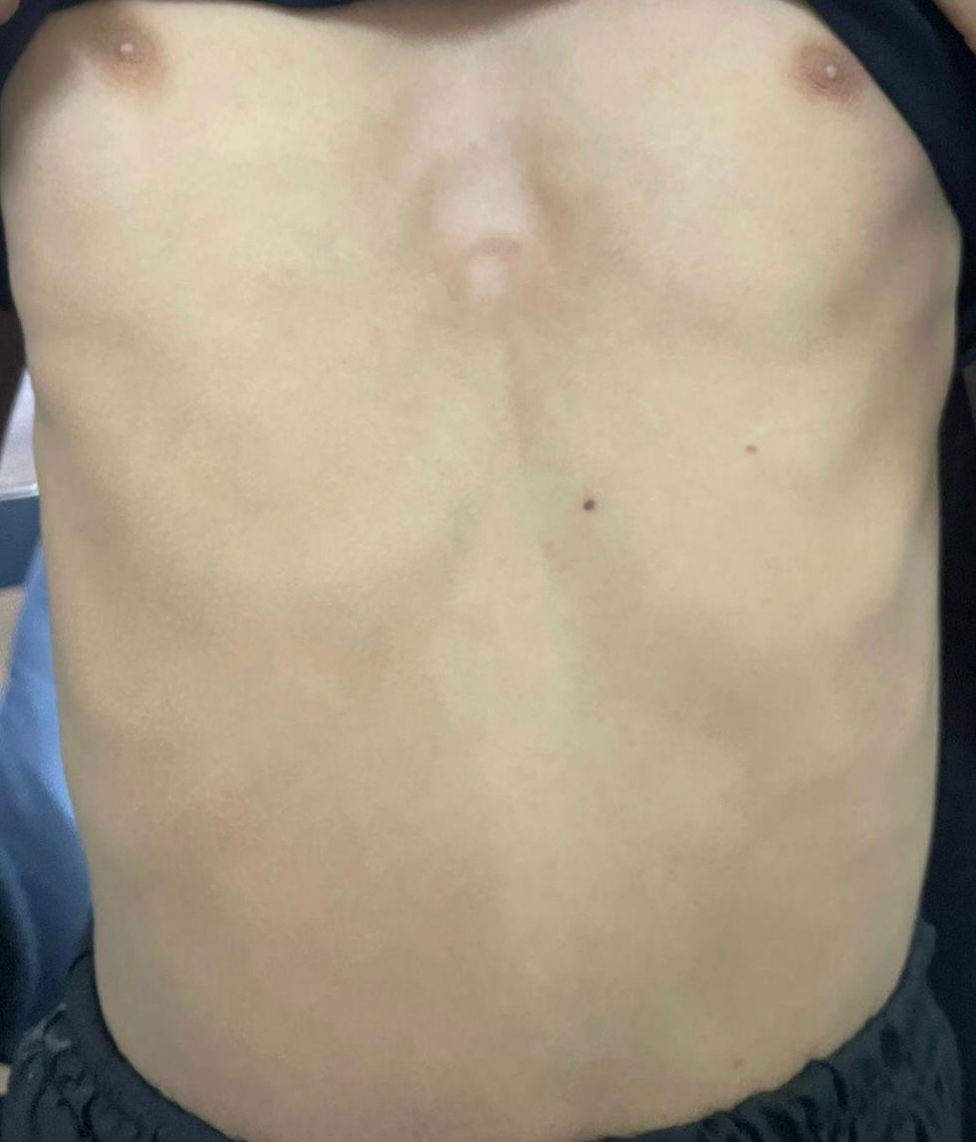
funnel chest appearance below the nipple over the xiphoid process

